# P01.46. Assessment of autonomic tone at rest and during meditation in a longitudinal study of an eight-week meditation intervention

**DOI:** 10.1186/1472-6882-12-S1-P46

**Published:** 2012-06-12

**Authors:** G Desbordes, R Barbieri, L Citi, S Lazar, L Negi, C Raison, E Schwartz

**Affiliations:** 1 Boston University, Boston, USA; 2 Massachusetts General Hospital, Boston, USA; 3 Emory University, Atlanta, USA; 4 University of Arizona, Tucson, USA

## Purpose

Measures of heart rate variability (HRV) and respiratory sinus arrhythmia (RSA), which are the healthy fluctuations in heart rate that reflect autonomic influences on cardiac activity, have been proposed as indicators of physical and psychological health. Previous studies suggest that HRV increases during some meditative states, but it is not clear how autonomic tone is affected either in the short term during meditative states, nor as a long-term result of meditation practice. Here we test two hypotheses: (1) eight weeks of meditation training will improve autonomic tone, in the form of increased overall HRV and decreased low-frequency HRV to high-frequency HRV (LF/HF) ratio; and (2) RSA increases during the meditation state compared to rest.

## Methods

We collected electrocardiogram recordings as part of a larger randomized controlled trial of the effects of an eight-week meditation training intervention on healthy adults without prior meditation experience. Here we report data from eight subjects before and after the “mindful-attention” meditation intervention and the control intervention. The recordings were performed while the subjects were lying supine, at rest (for both groups) and in a meditative state (for the meditation group only). We computed measures of HRV and RSA across a 3-minute epoch using a point-process model of heart beat dynamics.

## Results

We found a longitudinal increase in HRV and a longitudinal decrease in LF/HF ratio (i.e., a more predominant vagal tone), both consistent with improved autonomic function, in the meditation group but not in the control group. We also found a marked increase in RSA during meditation compared to rest.

## Conclusion

These preliminary outcomes, although derived from a small sample, support our hypotheses. They suggest that measures of RSA might be useful markers of the meditative state, and encourage further investigation on the efficacy of HRV measures to indicate a general improvement of autonomic health after meditation interventions.

